# Data Integration and Interoperability for Patient-Centered Remote Monitoring of Cardiovascular Implantable Electronic Devices

**DOI:** 10.3390/bioengineering6010025

**Published:** 2019-03-17

**Authors:** Carly Daley, Tammy Toscos, Michael Mirro

**Affiliations:** 1 Parkview Mirro Center for Research and Innovation, Parkview Health, 10622 Parkview Plaza Dr., Fort Wayne, IN 46845, USA; tammy.toscos@parkview.com (T.T.); michael.mirro@parkview.com (M.M.); 2 Department of BioHealth Informatics, IUPUI School of Informatics and Computing, 535 W. Michigan St., Indianapolis, IN 46202, USA; 3 Department of Medicine, Indiana University School of Medicine, 340 West 10th St., Indianapolis, IN 46202, USA

**Keywords:** cardiovascular implantable electronic device, CIED, remote monitoring, interoperability, data integration, data standards, patient-centered, patient engagement

## Abstract

The prevalence of cardiovascular implantable electronic devices with remote monitoring capabilities continues to grow, resulting in increased volume and complexity of biomedical data. These data can provide diagnostic information for timely intervention and maintenance of implanted devices, improving quality of care. Current remote monitoring procedures do not utilize device diagnostics to their potential, due to the lack of interoperability and data integration among proprietary systems and electronic medical record platforms. However, the development of a technical framework that standardizes the data and improves interoperability shows promise for improving remote monitoring. Along with encouraging the implementation of this framework, we challenge the current paradigm and propose leveraging the framework to provide patients with their remote monitoring data. Patient-centered remote monitoring may empower patients and improve collaboration and care with health care providers. In this paper, we describe the implementation of technology to deliver remote monitoring data to patients in two recent studies. Our body of work explains the potential for developing a patent-facing information display that affords the meaningful use of implantable device data and enhances interactions with providers. This paradigm shift in remote monitoring—empowering the patient with data—is critical to using the vast amount of complex and clinically relevant biomedical data captured and transmitted by implantable devices to full potential.

## 1. Introduction

Advancements in engineering and computer science have improved the capabilities of implantable medical devices and remote monitoring. In the shift toward a patient-centered health care model, there are new possibilities for how data from cardiovascular implantable electronic devices (CIEDs) can empower patients to confidently manage and engage in their health [1]. The current remote monitoring model is system-centered, and patients are provided little or no discrete data from their device. Our team was the first to report messaging patients’ device data in a discrete data format as the first step to testing patient-centered remote monitoring [2]. Further, data standardization and interoperability are needed to build technologies that can support interpretation of implantable device data by patients and providers. 

Notably, Cronin and Varma described remote monitoring as a “paradigm shift of the 21st century” [3] as it allows for improved efficacy and efficiency over in-clinic checks. However, remote monitoring of CIEDs includes proprietary data formats from multiple device manufacturers, creating challenges for data integration and interoperability. Integrating the Healthcare Enterprise (IHE) and the Heart Rhythm Society developed a technical framework for CIED data integration into diverse platforms in response to this issue [4]. Development of the framework has been an ongoing collaboration of clinical research scientists and industry biomedical engineers to develop an Institute of Electrical and Electronics Engineers (IEEE) data standard to allow structured data to flow into the electronic health record (EHR) environment with consistency between industry vendors. The framework not only supports data integration for improved patient safety and reduced clinical burden but also allows the delivery of remote monitoring CIED data to the patient in a personal health record (PHR) or similar platform. In this paper, we highlight the advancements in remote monitoring technology for CIED data and present an overview of two studies that pioneered the use of technology to provide remote monitoring CIED data directly to patients with implanted devices. This work represents a novel shift in the management of implantable medical device data from remote monitoring, placing patients at the center of their care and improving the accessibility of data for timely detection and intervention. 

### Remote Monitoring of Cardiovascular Implantable Electronic Devices

Cardiovascular implantable electronic devices (CIEDs) provide critical information such as indicators of device functioning and physiological diagnostic data. These data provide important information related to the device, such as battery and lead status, as well as alerts to deterioration in health status, such as arrhythmia and ventricular therapies. Until recently, patients with CIEDs were required to come in to the clinic for device checks. To improve efficiency, clinics implemented remote monitoring to collect, review, and evaluate the data from implanted devices between clinic checks. Remote monitoring technology allows for timely detection of device status, arrhythmias, inappropriate shocks, and episodes [5]. Enrolling patients in remote monitoring demonstrates improved survival and economic outcomes in patients after implant of CIEDs [6,7].

The number of patients with CIEDs is increasing, and the complexity of devices and data evolve with advances in technology. Remote monitoring can provide near real-time diagnoses and reduces time to treatment due to early detection. However, clinics still need to manage large data sets from device interrogations. The clinic staff reviews, triages, summarizes, and stores the data in an EHR for review by a clinician. For quality of care, if there are actionable data, the clinic must follow up with the patients in a timely manner, and if patients do not transmit on schedule, the clinic must contact the patients regarding missed transmissions. Despite the advantages and overall satisfaction with remote monitoring, patient adherence remains low [3,8]. Another challenge with managing remote monitoring data is the proprietary format of the data from multiple device vendors [4]. Managing the data involves integration using separate proprietary platforms and storing reports electronically in Portable Document Format (PDF) in the EHR. Thus, data are not available in a readily usable discrete data format [4,9]. 

With an increasing volume of health data, we need to use technology to better manage the data for patient safety and improved clinical workflow. We need a standardized way to integrate remote monitoring data into the EHR, with a standard set of observations, consistent across all types of devices. To address these issues of standardization and interoperability, Integrating the Healthcare Enterprise and the Heart Rhythm Society developed the Implantable Device Cardiac Observation (IDCO) profile, a standard for device data incorporating IEEE 11073-10103 nomenclature, Health Level-7 (HL7) messaging, and a technical framework [4]. The IDCO profile addresses the need for standardized observations to integrate seamlessly from all device vendors into various electronic platforms without human intervention. Over the past several years, research using the IDCO profile has demonstrated the potential for integrating data from at least one device manufacturer [9,10]. However, the field lacks research on using the data for a patient-centered purpose. In response to this need, we conducted two studies which utilized the IDCO profile to integrate data from St. Jude Medical into the EHR and directly to a patient platform. 

## 2. Patient-Centered Remote Monitoring 

In this section we present an overview of the design and development of two studies which utilized the IDCO profile and data from remote monitoring of St. Jude Medical implantable cardioverter defibrillators (ICDs) to provide patients with data from their devices. Both studies were approved by the Parkview Health Institutional Review Board. All participants signed informed consent forms in accordance with the Declaration of Helsinki before any study activities. 

### 2.1. Overview of Study Designs

#### 2.1.1. Study 1: Feasibility Study

A feasibility study took place in 2014 at Parkview Health in Indiana, in collaboration with St. Jude Medical, NoMoreClipboard, and WebChart. The study was funded by the Office of the National Coordinator for Health Information Technology, as part of the federal initiative to foster innovation in health information technology for patient-centered care and improved health outcomes. The study included the implementation of the IDCO profile to deliver messages from the St. Jude Medical remote monitoring database, Merln.net™ directly to the PHR, NoMoreClipboard. After establishing the data flow and the design and function of NoMoreClipboard to display device data, twenty-one participants with St. Jude Medical ICDs were enrolled in the study and followed for three months. At enrollment, participants were shown how to access and view their NoMoreClipboard PHR and took a survey that included demographic information and assessment of patient activation (Patient Activation Measure [11]). At the end of the three months, the research team administered a follow-up survey and conducted interviews with each participant to explore their experiences using the PHR to view their ICD data. Detailed methods are published elsewhere [12]. 

#### 2.1.2. Study 2: Evaluation 

Continuing in 2014, our research group began a larger six-month evaluation study at Parkview Health in collaboration with St. Jude Medical and Epic (MyChart® patient portal) to uncover patient perspectives about receiving their ICD data through MyChart® and on paper. This study was funded by St. Jude Medical and study demonstrated a similar data flow process as the feasibility study, using the IDCO profile in a different EHR/portal system. One hundred ninety-one patients with ICDs participated in this study across three groups: 1) participants randomized to receive their data electronically in MyChart®; 2) participants randomized to receive the same report on paper in the mail; 3) a self-selected group of participants who did not receive their data. Baseline, three-month, and six-month surveys were administered to all participants. Detailed methods are published elsewhere [13]. See Table 1 for a high-level comparison of study 1 and study 2.

### 2.2. Data Integration

**Figure 1:**
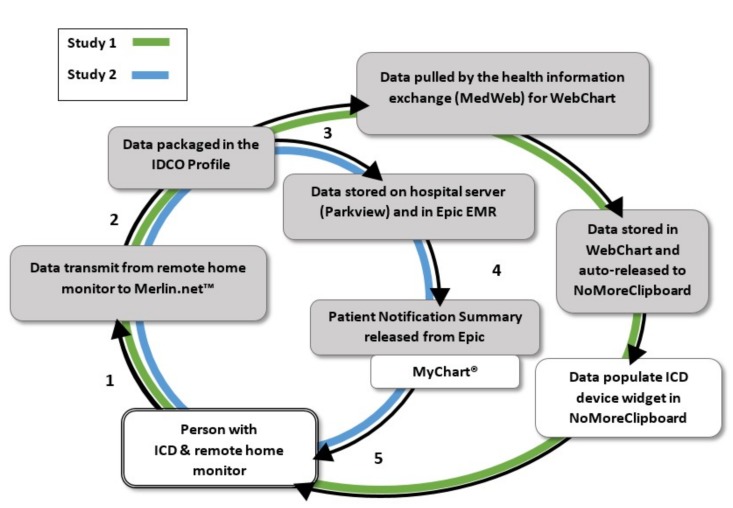
Pathway of remote monitoring implantable cardioverter defibrillator (ICD) data for study 1 and 2, starting with the patient’s ICD and returning back to the patient in a personal health record (NoMoreClipboard) or patient portal (MyChart®). Steps one and two were the same for both studies and diverged based on the electronic health record (EHR) and personal health record (PHR) or portal system. The white boxes indicate non- Health Insurance Portability and Accountability Act (HIPAA) regulated entities and gray boxes indicate HIPAA regulated entities.

For both studies, the process of delivering data from the device to a patient portal was developed per Health Insurance Portability and Accountability Act (HIPAA) regulations (Figure 1). Data from remote monitoring, stored in the St. Jude Medical remote monitoring database (Merlin.net™) were exported to Parkview’s server. Developers installed Cloverleaf® Secure Courier, a software that provides secure messaging for healthcare organizations and disparate systems, before establishing connectivity from Merlin.net™ to the server. Per standard of care, the clinic viewed patients’ transmissions in Merlin.net™ and processed reports using the third party software, Paceart™, to integrate the data into a PDF document and store in the EHR. At the time of the feasibility study, Parkview Health was newly implementing Epic as the primary EHR and another EHR system, WebChart, was still partially active in the cardiology clinic.

For the feasibility study, patient transmissions from remote monitoring were exported to WebChart, using a secure transmission and specialized data mailboxes to match the messages to patients’ charts in WebChart. At each patient enrollment in the study, research staff manually exported Merlin.net™ data from Webchart to the patient’s NoMoreClipboard account. This manual transmission initiated the flow of data for future transmissions so that patients would receive their remote monitoring transmissions directly to their PHR in near real time. 

For the larger evaluation study, Epic and St. Jude Medical collaborated with Parkview and the research team to build a Patient Notification Summary in Epic’s MyChart®. Similar to WebChart and NoMoreClipboard, Epic received messages from Merlin.net™ so that remote monitoring transmissions would create a message in the patients’ charts. The development included a report template that would display the data. Testing ensured that the transmission data would show up as a lab report in the patients’ MyChart® account when the report was manually released from the EHR to MyChart®. 

### 2.3. PHR and Portal Interface Development

The research team worked with NoMoreClipboard and Epic to create the PHR interfaces for both studies, respectively. NoMoreClipboard developers built a widget (“Implantable Cardiac Device”) in the PHR to display the data for the feasibility study and Epic (MyChart®) developers utilized the standard report format to create a patient report, called the ICD Patient Notification Summary for the evaluation study. The NoMoreClipboard widget appeared on the home page of the PHR with a link to access a large set of data available from IDCO profile. The widget display included the number of shocks and antitachycardia pacing therapies (ATPs), lead impedance values and battery status (measured as months remaining to replacing battery intervals), and indicators (such as a green check) to display normal status. These three major display components were identified as high-value data elements with guidance from the Heart Rhythm Society and clinical experts. Patients could view additional data elements by following a link embedded in the widget, including lead information, lead status and measurement details, interrogation date, time, and type, whether or not the device was re-programmed during the session, name of clinic, battery measurements (date and time, status, voltage, longevity remaining, recommended replacement time trigger), capacitor charge type, last capacitor charge date and time, time to charge capacitor, energy to charge capacitor, lead channel measurements, sensing polarity, location of anodes and cathodes, bradycardia and tachycardia settings and rates, and other statistics. 

**Figure 2:**
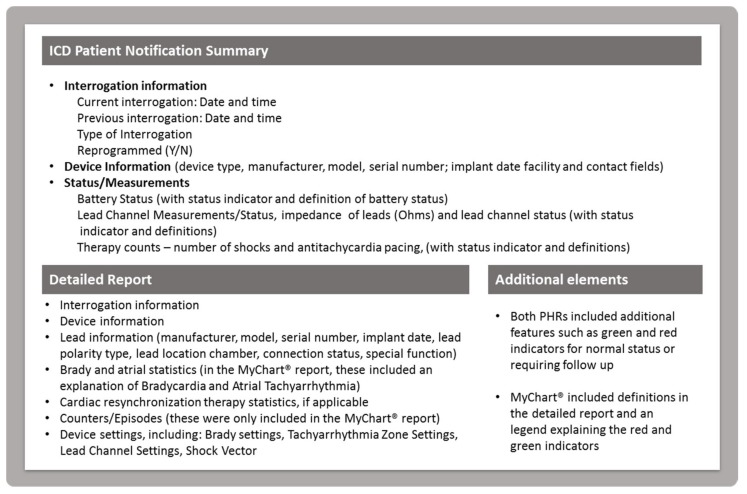
The image displays the categories of data elements provided to participants from implanted devices. There were variations in the data fields based on the type of ICD and relevant statistics. This image describes the content and does not portray the actual display of the PHR or portal.

For the MyChart® ICD Patient Notification Summary implemented in the evaluation study, developers adjusted the report template to show the three major components (number of shocks, number of ATPs, and battery status) in the top section, and then patients could view the additional data elements by scrolling down the page. Additionally, Epic added green and red indicators for data above or below threshold (to indicate, for example, that the leads were above or below the impedance level threshold for lead integrity) as well as an explanation of terms written directly on the report (such as what impedance means). See Figure 2 for an overview of content included in the widget display and ICD Patient Notification Summary. Images of the study-specific interfaces are published elsewhere [12,13].

Challenges to connecting the data from the IDCO profile included ease of understandability. In an effort to provide more clarity, the ICD Patient Notification Summary in study 2 included more definitions on the report, such as a definition of bradycardia and explanation of “RV” (right ventricle). In this work, data need to be carefully mapped to the display to ensure accuracy, and the display requires design and development for patients to be able to read and understand with ease. This requires further development of the IDCO profile to ensure all data elements from CIEDs are standardized so they can be used as needed in meaningful displays. While study 1 and study 2 both demonstrated the feasibility of delivering the data from remote monitoring to a PHR or patient portal platform, challenges of presenting the data in a meaningful way require in-depth research into data mapping and design of displays. 

### 2.4. Overview of Study Results

#### 2.4.1. Study 1: Feasibility Study

Twenty-one participants were enrolled in the feasibility study. Participants were predominantly male (76.2%), age 62 or older (81.0%) All participants were white, with the exception of one participant who did not indicate race, and who selected Hispanic or Latino ethnicity. Remote monitoring experience ranged from less than one year (*n* = 9) to over five years (*n* = 3). All participants received at least one transmission to their NoMoreClipboard PHR with a maximum of three during the study period. The qualitative analysis of patient interviews revealed that participants perceived potential benefits from receiving their data, such as feedback that their remote monitor was working. Although some participants were interested in receiving their data, several felt that the report had too much information, was too complex, and was not easy to understand. While some were interested in the detailed data so they could track their battery status and pacing, for example, others were content without any specific data elements from their device. The results suggested that the presentation of the data or feedback from remote monitoring should be tailored to the individual patient [12]. 

#### 2.4.2. Study 2: Evaluation Study

Of the 144 participants in Groups A and B, 16 did not receive a patient notification summary because they did not have any remote transmissions during the study period. Therefore, the analysis includes 128 participants who received a summary in Group A (*n* = 62) and Group B (*n* = 66). The participants were predominantly male (68.8%), age 66 or older (57.5%) and white (93.0%). Participants had been on remote monitoring for a mean of 3.2 years, ranging from new (<1 year) to in their 6th year (*n* = 119; remote monitoring history was unavailable for 9 participants). A mean of 1.7 patient notification summaries per participant were sent during the study period, (minimum 1 and maximum 8). Overall, participants preferred the ICD summary over the current standard of care which involved a letter from the clinic indicating their remote monitoring ICD check status [13]. 

## 3. Discussion 

Our work has initiated a paradigm shift in remote monitoring of CIEDs by delivering ICD data directly to patients through a PHR. The engineering advancement in CIED functionality, as well as performance, can be used to further optimize care by sharing actionable data with patients as a means to improve clinical outcomes. Our findings suggest that it is not only feasible to deliver data from remote monitoring directly to patients, but also that patients perceive a benefit from having access to their remote monitoring data. A recent study examined the use of a technology probe with 10 ICD patients to understand patients’ perceptions about taking a more active instead of passive role in home monitoring to manage symptoms. Overall, patients wanted to become more active in their health management and interpret data collaboratively with their health care providers. Participants were uncertain about their role in home monitoring, suggesting that more studies testing the use of real data from remote monitoring should be conducted [14]. The vision to engage patients directly with the remote monitoring process may enhance clinical and economic outcomes by helping patients take an active role in managing their health condition. 

The two studies described in this paper show the technical feasibility of delivering remote monitoring ICD data to patients. The strengths of this work include the implementation of a robust technical framework and an in-depth evaluation of patient experiences. The limitations of this work include the limited number of patient notification summaries that patients received, which was associated with remote monitoring transmission frequency in the three-month and six-month study periods. While there were positive responses to receiving data, the participants in these studies had access to technology and volunteered to participate. Therefore, the perceptions of people who were not adherent to remote monitoring, who may not be interested in or are opposed to receiving data, and/or do not have access to technology, were not represented. The study samples were comprised of predominantly educated, white, individuals, therefore, do not represent the diverse population of people with implanted devices.

The next step is to test the impact of these interventions on a larger scale to evaluate targeted outcomes and the resulting implications for patient self-care and the clinical management of patients living with CIEDs. Despite the substantial benefits of remote monitoring, patient participation and adherence remain low [15]. Thus, to realize the full potential of the sophisticated engineering in CIEDs, we need to understand a way to meaningfully engage patients, and we believe this can be done by presenting meaningful data to patients as feedback from remote monitoring. The authors of a recent review of CIED use offer helpful suggestions that may lead to improved engagement including standardizing transmission data, increasing the frequency of comprehensive information, and using predictive algorithms with the data as a means to prevent worsening conditions [15]. 

Continued research should be aimed at determining the feasibility and benefits of integrating data from multiple vendors in a structured format, which will reduce the need for multiple proprietary formats. The major CIED vendors need to consistently use the common data standards to avoid “data-blocking” that present barriers to clinics and patients accessing data that can be an aid in the successful management of their disease. Currently, our team is pursuing new research examining the implementation of platforms that integrate data from multiple vendors, and there are ongoing efforts in support of this pursuit. For example, a clinical trial in Poland is testing a platform which integrates data from three device manufacturers using the IDCO profile, comparing remote monitoring to the standard of care on all-cause death or hospitalization due to cardiovascular reasons as the primary endpoint [16]. Previous work has demonstrated the effective utilization of the IDCO profile for data communication [9,10,17], and we now need to test the impact on patient outcomes (quality of life, engagement in health, remote monitoring adherence, and clinical metrics related to disease state). 

Another important aspect of the paradigm shift is presenting data to patients in a way that adds value to their self-care routines. Lessons learned from the two studies described in this paper are that patients are seeking tailored, meaningful ways of viewing and understanding their health data [12,13]. In follow up to the studies described in this paper, our lab has begun investigating patient needs using patient-centered design methods, including focus groups and participatory design towards building an actionable, patient-facing view of the data [18,19,20]. User-centered design methods are critical to develop a tool that meets patient needs and provides the necessary scaffolding for self-management.

## 4. Conclusions

In this paper we propose a shift in the current practice of remote monitoring of CIED data, moving from a predominantly proprietary system to one with greater interoperability and a patient-centered focus. To accomplish this, we must provide a standardized, structured data format in order to:

- Improve clinician workflow and accessibility to data;
- Provide patients with a meaningful, secure technology platform to view their data.

These objectives will provide a clinical environment that will allow for an evaluation of the ways in which patient-centered remote monitoring impact quality of life, engagement in health, remote monitoring adherence, and clinical metrics related to disease state. The ultimate goal of this work is to provide a universal data set and meaningful presentation of data from CIEDs that patients can easily read, understand, and use. At present, this work will require considerable effort to provide customized and tailored messaging to patients based on cultural preferences, educational level, and other factors. Using a technical framework for data interoperability has the potential to unlock access to life-saving biomedical diagnostic data, releasing the untapped potential of CIEDs for improving patient health outcomes. This work is transformational for cardiac care, closing a feedback loop in remote monitoring that empowers patients to better manage their cardiac condition. Current work has yet to demonstrate if this approach can impact clinical and economic outcomes. Future studies should focus on exploring these relationships.

## Figures and Tables

**Table 1:** Overview of study 1 and study 2 designs and number of transmissions sent to a personal health record (PHR) or portal during the studies.

	Study 1	Study 2
Study Type	Feasibility	Evaluation
N	21	191
Study groups	N/A	Group A: Receive Patient Notification Summary in MyChart® (*n* = 73) Group B: Receive Patient Notification Summary on paper in the mail (*n* = 71) Group C: Self-selected group which did not receive a Patient Notification Summary (*n* = 47)
Participant study duration	3 months	6 months
Device manufacturer	St. Jude Medical	St. Jude Medical
EHR	Webchart	Epic
PHR/portal	NoMoreClipboard	MyChart®
Data collection	Chart review Patient interviews and surveys Provider surveys	Chart review Patient surveys Provider surveys
Transmission frequency	Remote scheduled (3 months) Alert basis Patient initiated basis	Remote scheduled (3 months) Alert basis Patient initiated basis
Total number of transmissions sent to PHR/portal during the study	30	215 MyChart® and paper reports combined
Average number of transmissions sent to PHR/portal during the study	1.4 (min 1, max 3)	1.7 MyChart® and paper reports combined (min 1, max 8)

## Abbreviations

CIED	Cardiovascular Implantable Electronic Device
IHE	Integrating the Healthcare Enterprise
IEEE	Institute of Electrical and Electronics Engineers
EHR	Electronic Health Record
PHR	Personal Health Record
PDF	Portable Document Format
IDCO	Implantable Device Cardiac Observation
ICD	Implantable Cardioverter-Defibrillator
HIPAA	Health Insurance Portability and Accountability Act
